# Association of the atherogenic index of plasma and other lipid indices with abnormal glucose metabolism in patients with ischemic stroke

**DOI:** 10.3389/fendo.2025.1677653

**Published:** 2025-10-06

**Authors:** Ting Liu, Weizhen Li, Shiwen Zhang, Ye Wang, Xin Zou, Yiran Zhao, Jiawen Dong, Lin Zhu, Shencheng Luo, Bernhard Kolberg, Jing Li

**Affiliations:** ^1^ First Teaching Hospital of Tianjin University of Traditional Chinese Medicine, Tianjin, China; ^2^ National Clinical Research Center for Chinese Medicine Acupuncture and Moxibustion, Tianjin, China; ^3^ Department of Internal Medicine, Mannheim Medical School of Heidelberg University, Mannheim, Germany

**Keywords:** ischemic stroke, atherogenic index of plasma, lipid indices, pre-DM, T2DM

## Abstract

**Objective:**

To evaluate the relationship of the atherogenic index of plasma (AIP) and other lipid indices with prediabetes mellitus (Pre-DM) and type 2 diabetes mellitus (T2DM) in patients with ischemic stroke (IS), with a focus on exploring the clinical application value of AIP in the assessment of glucose metabolism disorders.

**Methods:**

This study was conducted for the first time based on a large-scale IS cohort. 18,604 patients with IS were enrolled and subjected to comprehensive lipid profile assessments. The correlation between AIP and other commonly used lipid indices was systematically compared. Based on glucose levels, the patients were divided into three groups: normal glucose (NG), Pre-DM, and T2DM. The relationship of AIP and other lipid indices with Pre-DM and T2DM was evaluated through multivariable logistic regression. Furthermore, the dose-response correlation of AIP with Pre-DM and T2DM across varying genders and age groups was explored through the Restricted Cubic Spline (RCS) model, which was employed to analyze non-linear associations between variables.

**Results:**

We found that triglyceride (TG), total cholesterol (TC)/high-density lipoprotein cholesterol (HDL-C), low-density lipoprotein cholesterol (LDL-C)/HDL-C, TG/HDL-C, non-HDL-C, remnant cholesterol (RC), RC/HDL-C, and AIP were all positively correlated with Pre-DM and T2DM. Notably, AIP demonstrated the highest specificity in this context. AIP was then divided into tertiles, with the T3 group showing the strongest correlation with both conditions compared with the T1 group. The correlation was stronger among females and patients aged ≥60 years. RCS analysis further indicated a non-linear positive dose-response relationship between AIP and T2DM across all genders and ages.

**Conclusion:**

In IS patients, AIP exhibits a stronger association with Pre-DM and T2DM than other lipid indices, especially in female patients and those aged 60 years and above.

## Graphical Abstract

Pre-DM, prediabetes mellitus; T2DM, type 2 diabetes mellitus; AIP, atherogenic index of plasma; IS, ischemic stroke; TC, total cholesterol; TG, triglycerides; LDL-C, low-density lipoprotein cholesterol; HDL-C, high-density lipoprotein cholesterol; RC, remnant cholesterol.

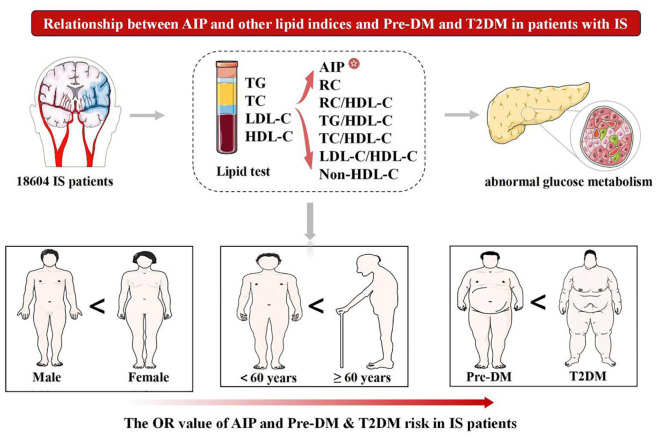

## 1 Introduction

Metabolic disorders, particularly dyslipidemia and hyperglycemia, can significantly increase the risk of stroke (1, 2). Type 2 diabetes mellitus (T2DM) is a well-established independent risk factor for ischemic stroke (IS). Even mild glucose metabolism abnormalities, such as prediabetes mellitus (Pre-DM), significantly increase the risk of IS (3). Such abnormal glucose metabolism states not only impair multiple organs, including the kidneys and heart, but also directly participate in the pathological process of cerebrovascular diseases (4). Consequently, it is crucial to strengthen the management of glucose metabolism abnormalities, accurately identify high-risk populations, and simultaneously explore reliable and efficient novel biomarkers. The atherogenic index of plasma (AIP), as an index that is simple to calculate and low in cost, shows considerable potential.

Currently, a variety of unconventional lipid indices, such as total cholesterol (TC)/high-density lipoprotein cholesterol (HDL-C), triglycerides (TG)/HDL-C, low-density lipoprotein cholesterol (LDL-C)/HDL-C, non-HDL-C, remnant cholesterol (RC), RC/HDL-C, and AIP, are strongly linked to Pre-DM and T2DM (5–7). Among them, AIP, calculated as log (TG/HDL-C), can effectively predict the size of lipoprotein particles and is associated with insulin resistance (IR) (8). In terms of identifying abnormal glucose metabolism, it exhibits superior predictive value compared to conventional lipid indicators like TC and TG (9). However, the popularity of AIP in clinical practice and health screening is still far lower than that of conventional lipid indicators. In contrast, although small dense low-density lipoprotein cholesterol (sdLDL-C) is associated with diabetes and acute IS, its detection process is complex and costly, which limits its clinical application (10). This further highlights the advantages of AIP and similar indicators in terms of practicality and economic benefits.

Researchers have shown that AIP can influence the prevalence of Pre-DM and T2DM, especially among females (11). The CHARLS cross-sectional study also uncovered a nonlinear and positive correlation of AIP with the occurrence of Pre-DM and T2DM (12). Nevertheless, previous studies have mainly focused on patients with coronary heart disease or the general population (13); for IS patients, studies investigating the correlation between AIP (and other lipid indices) and the progression of Pre-DM and T2DM are significantly insufficient, and relevant evidence remains scarce.

This study, for the first time, investigated the association between lipid indices such as AIP and Pre-DM and T2DM in 18,604 patients with IS, conducting stratified analysis based on gender and age differences, aiming to identify potential biomarkers with clinical value for the early risk warning in the IS population.

## 2 Methods

### 2.1 Study population

**Figure 1:**
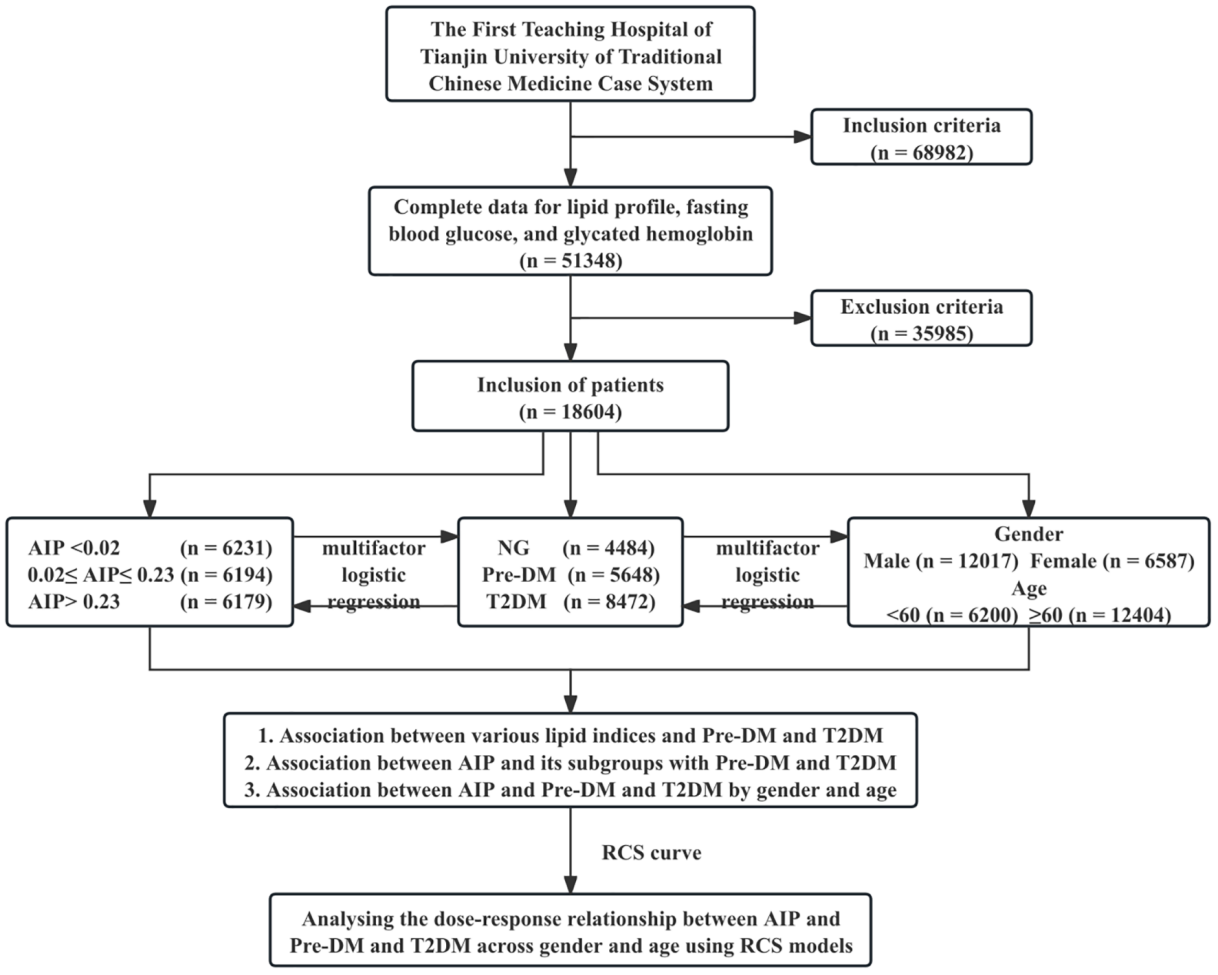
Study flowchart. NG, normal glucose; Pre-DM, prediabetes mellitus; T2DM, type 2 diabetes mellitus; AIP, atherogenic index of plasma; RCS, Restricted Cubic Spline.

In this retrospective study, we enrolled 68,982 patients diagnosed with IS at the First Teaching Hospital of Tianjin University of Traditional Chinese Medicine from January 1st, 2013, to May 1st, 2023. The consecutive enrollment method was used to recruit eligible patients in order to minimize the potential impact of selection bias on the study results. Rigorous exclusion criteria were implemented in subject selection to ensure the precision of the study’s findings. **Figure 1** shows a flowchart of the patient selection procedure. This was registered with the Chinese Clinical Trial Registry (registration number: ChiCTR2100045415). The study was approved by the Ethics Committee of the First Teaching Hospital of Tianjin University of Traditional Chinese Medicine (approval number: TYLL2020(K) 057).

### 2.2 Criteria

#### 2.2.1 Criteria for inclusion

Patients who met the diagnostic criteria of Chinese Guidelines for the Diagnosis and Treatment of Acute Ischemic Stroke (2018) (14): i) Acute onset of the disease; ii) Presence of varying degrees of neurological deficits; iii) Imaging examinations confirmed the lesions responsible for symptoms/signs lasting more than 24 hours; iv) non-vascular pathologies were excluded; v) Cerebral hemorrhage was ruled out by computed tomography (CT) or magnetic resonance imaging (MRI). Furthermore, all clinical information was complete and traceable.

#### 2.2.2 Criteria for exclusion

Participants with space-occupying lesions, brain abscesses, or cerebral hemorrhages; those with a background of infectious diseases, cancerous tumors, or severe liver and kidney diseases; those diagnosed with elderly IS or with a history of IS; those who received thrombolysis or thrombectomy during the acute phase of IS; those whose lipid indices, other pertinent information, or statistics were inaccurate; and those who used any lipid-lowering and glucose-lowering drugs.

### 2.3 Data collection

Demographic information was collected using a standard structured questionnaire, including age, gender, lifestyle data (such as drinking status and smoking status), medical history, and medication information. Smokers were defined as individuals who have smoked at least 100 cigarettes in their lifetime (15); drinkers were defined as those who consumed alcohol at least once a week (16). The patient’s systolic blood pressure (SBP) and diastolic blood pressure (DBP) were measured by skilled professionals using automated blood pressure measuring devices (Yuwell Medical Equipment & Supply Co, Ltd., Jiangsu, China). Hypertension was defined as DBP ≥90 mmHg or SBP ≥140 mmHg (17) or present antihypertensive drug use. T2DM was defined as an elevated hemoglobin A1c (HbA1c) level to ≥6.5% (18). Pre-DM was defined as an abnormal glucose homeostasis state characterized by impaired fasting glucose, impaired glucose tolerance, or both (19). Diabetes status was categorized into normal glucose (NG) (HbA1c <5.7% or fasting blood glucose (FBG) <5.6 mmol/L), Pre-DM (5.7≤ HbA1c ≤6.4% or 5.6≤ FBG ≤6.9 mmol/L), and T2DM (HbA1c ≥6.5% or FBG ≥7.0 mmol/L) (18). Notably, all individuals in the NG group had never used glucose-lowering drugs. The National Institutes of Health Stroke Scale (NIHSS) score, which ranges from 0 to 42 points, was utilized to assess the severity of IS upon admission, with higher scores indicating more serious strokes (20). Venous blood samples were collected from each participant in the morning after at least 8 hours of fasting. An automated blood analyzer (Hitachi, Ltd., Chiyoda, Tokyo, Japan) was employed to assess other indicators, such as TC, TG, LDL-C, HDL-C, glucose (Glu), and HbA1c. Unconventional lipid indices were computed from conventional lipid indicators using the following formulas: TC/HDL-C, TG/HDL-C, LDL-C/HDL-C, non-HDL-C=TC - HDL-C, RC=non-HDL-C - LDL-C, RC/HDL-C, and AIP=log (TG/HDL-C).

### 2.4 Quality control

The inclusion and exclusion criteria were strictly followed during the data collection process; an intelligent platform was utilized to screen patient data. A rigorous quality control was implemented to ensure accuracy and completeness: two researchers reviewed medical records, data entry personnel double-checked, and two independent researchers conducted a second review.

### 2.5 Statistical analysis

For continuous variables, normality tests were first performed. Variables that conformed to normal distribution were expressed as mean ± standard deviation (Mean ± SD); non-normally distributed variables were presented as the median and interquartile range (IQR). For between-group comparisons, the nonparametric Kruskal-Wallis H test was applied in case of skewed continuous variables, whereas the chi-square (χ2) test was used for categorical variables. The P-trend was calculated by modeling the median of their tertiles as a constant variable, and the strength odds ratio (ORs) and 95% confidence intervals (CIs) were determined through multivariate logistic regression to explore the correlations between Pre-DM and T2DM. In the multivariate regression, the variance inflation factor (VIF) method was applied to assess the multicollinearity between variables to ensure the robustness of the model. Moreover, age, gender, drinking, smoking, SBP, DBP, and antihypertensive drugs were considered potential confounders. Using Restricted Cubic Spline (RCS) models, we performed an in-depth analysis of the dose-response relationship of AIP with Pre-DM and T2DM, stratified by gender and age. Model fitting was optimized based on the Akaike Information Criterion (AIC). The optimal number of knots was determined by integrating clinical relevance, referring to the validated knot distribution from previous studies on the association between AIP and glucose metabolism disorders, and further considering the data characteristics of this study, along with the tertiles of AIP. The median of AIP was set as a reference point to assess the trend changes in Pre-DM and T2DM when AIP levels deviated from this reference point. All statistical analyses were performed using R software (version 4.4.1) and SPSS 27.0 (IBM Corp, Armonk, NY, USA). Differences were considered statistically significant with a two-sided test of *P*<0.05.

## 3 Results

### 3.1 Baseline characteristics

Among the 18,604 patients, 12,404 (66.7%) were aged 60 and older and 12,017 (64.6%) were male. The prevalence of Pre-DM was 30.3% (5,648), and that of T2DM was 45.5% (8,472). Participants were categorized into three groups based on glycemic status: NG, Pre-DM, and T2DM. The unconventional lipid indices in patients of the Pre-DM and T2DM groups were both higher than those in the NG group (**Table 1**).

**Table 1:** General characteristics of study populations based on the glycation levels.

Characteristic	Total (N=18,604)	NG (N=4,484)	Pre-DM (N=5,648)	T2DM (N=8,472)	*P*
Gender, n (%)					<0.001
Male	12017 (64.6)	3134 (69.9)	3549 (62.8)	5334 (63.0)	
Female	6587 (35.4)	1350 (30.1)	2099 (37.2)	3138 (37.0)	
Age, years	64.0 (57.0-72.0)	63.0 (55.0-71.0)	65.0 (58.0-73.0)	64.0 (57.0-71.0)	<0.001
DBP, mmHg	86.0 (79.0-94.0)	88.0 (79.0-96.0)	86.0 (78.0-94.0)	86.0 (79.0-94.0)	<0.001
SBP, mmHg	147.0 (134.0-161.0)	145.5 (132.0-160.0)	145.5 (133.0-160.0)	148.0 (135.0-162.0)	<0.001
NIHSS score	4.0 (2.0-7.0)	4.0 (2.0-7.0)	4.0 (2.0-7.0)	5.0 (2.0-7.0)	<0.001
Glu, mmol/L	5.75 (4.95-7.46)	4.87 (4.52-5.27)	5.34 (4.89-5.95)	7.56 (6.25-9.36)	<0.001
HbA1c,%	6.3 (5.7-7.6)	5.4 (5.2-5.5)	6.0 (5.8-6.2)	7.8 (7.0-8.9)	<0.001
TC, mmol/L	3.85 (3.14-4.70)	3.86 (3.20-4.63)	3.93 (3.24-4.74)	3.77 (3.06-4.71)	<0.001
TG, mmol/L	1.29 (0.96-1.76)	1.16 (0.88-1.58)	1.24 (0.93-1.70)	1.39 (1.05-1.88)	<0.001
LDL-C, mmol/L	2.22 (1.68-2.90)	2.24 (1.73-2.87)	2.27 (1.74-2.94)	2.17 (1.62-2.89)	<0.001
HDL-C, mmol/L	0.98 (0.82-1.17)	1.03 (0.87-1.23)	1.01 (0.85-1.20)	0.94 (0.79-1.12)	<0.001
Smoking, n (%)	9,293 (50.0)	2,420 (54.0)	2,823 (50.0)	4,050 (47.8)	<0.001
Drinking, n (%)	7,665 (41.2)	2,036 (45.4)	2,299 (40.7)	3,330 (39.3)	<0.001
Hypertension, n (%)	12,717 (68.4)	2,814 (62.8)	3,934 (69.7)	5,969 (70.5)	<0.001
Antihypertensive drugs, n (%)	12,276 (66.0)	2,733 (61.0)	3,757 (66.5)	5,786 (68.3)	<0.001
TG/HDL-C	1.33 (0.92-1.93)	1.13 (0.80-1.66)	1.25 (0.86-1.82)	1.50 (1.05-2.13)	<0.001
TC/HDL-C	3.88 (3.25-4.68)	3.71 (3.10-4.43)	3.85 (3.23-4.65)	4.02 (3.35-4.84)	<0.001
LDL-C/HDL-C	2.27 (1.73-2.90)	2.18 (1.67-2.75)	2.26 (1.73-2.88)	2.33 (1.78-2.99)	<0.001
RC	0.55 (0.37-0.79)	0.51 (0.34-0.71)	0.55 (0.37-0.78)	0.58 (0.40-0.82)	<0.001
RC/HDL-C	0.56 (0.37-0.83)	0.48 (0.31-0.71)	0.55 (0.35-0.81)	0.62 (0.41-0.91)	<0.001
Non-HDL-C	2.83 (2.21-3.61)	2.81 (2.20-3.47)	2.88 (2.27-3.64)	2.80 (2.17-3.66)	<0.001
AIP	0.12 (-0.04-0.29)	0.05 (-0.10-0.22)	0.10 (-0.07-0.26)	0.18 (0.02-0.33)	<0.001

Data are presented in the form of median (interquartile range) or count (percentage,%); the *P*-value was determined using the Kruskal-Wallis test or the chi-square test; NG, normal glucose levels; Pre-DM, prediabetes mellitus; T2DM, type 2 diabetes mellitus; DBP, diastolic blood pressure; SBP, systolic blood pressure; NIHSS, National Institutes of Health Stroke Scale; Glu, glucose; HbA1c, glycosylated hemoglobin; TC, total cholesterol; TG, triglycerides; LDL-C, low-density lipoprotein cholesterol; HDL-C, high-density lipoprotein cholesterol; RC, remnant cholesterol; AIP, atherogenic index of plasma.

### 3.2 Analysis of the association of various lipid indices with pre-DM and T2DM

TG and other unconventional lipid indices were positively correlated with the occurrence of Pre-DM and T2DM, while HDL-C showed a negative correlation with them. Among the lipid indices associated with Pre-DM and T2DM in patients with IS, AIP was the main associated factor (Pre-DM: OR: 1.96, 95% CI: 1.67-2.30, *P*<0.001; T2DM: OR: 6.99, 95% CI: 6.01-8.15, *P*<0.001) (**Table 2**).

**Table 2:** Analysis of the association of various lipid indices with Pre-DM and T2DM.

Lipid indices	Pre-DM		T2DM	
	OR (95% CI)	*P*	OR (95% CI)	*P*
TC, mmol/L	1.05 (1.02-1.09)	0.004	0.99 (0.96-1.02)	0.586
TG, mmol/L	1.22 (1.15-1.29)	<0.001	1.49 (1.42-1.57)	<0.001
LDL-C, mmol/L	1.04 (0.99-1.08)	0.094	0.96 (0.93-1.00)	0.078
HDL-C, mmol/L	0.74 (0.64-0.84)	<0.001	0.27 (0.24-0.31)	<0.001
Non-HDL-C	1.09 (1.05-1.13)	<0.001	1.08 (1.05-1.12)	<0.001
TC/HDL-C	1.16 (1.11-1.20)	<0.001	1.33 (1.28-1.37)	<0.001
TG/HDL-C	1.19 (1.14-1.25)	<0.001	1.46 (1.40-1.52)	<0.001
LDL-C/HDL-C	1.12 (1.07-1.18)	<0.001	1.25 (1.19-1.30)	<0.001
RC	1.54 (1.39-1.71)	<0.001	1.95 (1.77-2.15)	<0.001
RC/HDL-C	1.55 (1.41-1.71)	<0.001	2.19 (2.00-2.40)	<0.001
AIP	1.96 (1.67-2.30)	<0.001	6.99 (6.01-8.15)	<0.001

Pre-DM, prediabetes mellitus; T2DM, type 2 diabetes mellitus; TC, total cholesterol; TG, triglycerides; LDL-C, low-density lipoprotein cholesterol; HDL-C, high-density lipoprotein cholesterol; RC, remnant cholesterol; AIP, atherogenic index of plasma; OR, Odds ratio; CI, Confidence interval.

### 3.3 Association of AIP with pre-DM and T2DM

#### 3.3.1 Association of AIP with pre-DM and T2DM in total study participants

In the unadjusted Model a, the continuous variable AIP was significantly associated with Pre-DM and T2DM. In Model b adjusted for confounding factors, the risks of Pre-DM and T2DM increased by 2.61 times and 8.99 times, respectively. After categorizing AIP into tertile groups (T1: <0.02; T2: 0.02 - 0.23; T3: >0.23), the association between AIP and the risk of Pre-DM and T2DM was stronger in the T2 and T3 groups than in the T1 group (all *P*<0.001). Once the continuous AIP was converted into categorical variables, higher tertiles of AIP were associated with an increased likelihood of Pre-DM and T2DM (*P* for trend <0.001) (**Table 3**).

**Table 3:** Association of AIP with Pre-DM and T2DM.

Variables	Pre-DM		T2DM	
	OR (95% CI)^a^	OR (95% CI)^b^	OR (95% CI)^a^	OR (95% CI)^b^
AIP	1.96 (1.67-2.30)^*^	2.61 (2.20-3.08)^*^	6.99 (6.01-8.15)^*^	8.99 (7.66-10.54)^*^
T1	Reference	–	–	–
T2	1.23 (1.12-1.35)^*^	1.34 (1.22-1.47)^*^	1.94 (1.78-2.12)^*^	2.10 (1.92-2.29)^*^
T3	1.46 (1.32-1.61)^*^	1.70 (1.53-1.88)^*^	3.09 (2.82-3.39)^*^	3.51 (3.19-3.86)^*^
P-trend	<0.001	<0.001	<0.001	<0.001

T1: AIP <0.02, T2: 0.02≤ AIP ≤0.23, T3: AIP >0.23.

a Model a: unadjusted.

b Model b: gender, age, SBP, DBP, smoking, drinking, and antihypertensive drugs; ^*^ *P*<0.001.

Pre-DM, prediabetes mellitus; T2DM, type 2 diabetes mellitus; AIP, atherogenic index of plasma; OR, Odds ratio; CI, Confidence interval.

#### 3.3.2 Association of AIP with pre-DM and T2DM stratified by gender and age

Subgroup analysis results revealed that after adjusting for confounding factors, the levels of AIP as a continuous variable were associated with the incidence of Pre-DM and T2DM across different gender and age strata (*P*<0.01). Specifically, females had a higher OR than males in the Pre-DM and T2DM; elderly patients (age ≥60 years) exhibited markedly higher OR compared to younger patients (age <60 years). When AIP was grouped by tertiles with the T1 group as the reference, the disease risk in the T3 group was significantly increased. This risk elevation was particularly more pronounced in female diabetic patients and the elderly population (*P<*0.001) (**Table 4**).

**Table 4:** Association of AIP with pre-DM and T2DM among patients of varying genders and ages.

Gender/Age	Variables	Pre-DM		T2DM	
		OR (95% CI)^a^	OR (95% CI)^b b’^	OR (95% CI)^a^	OR (95% CI)^b b’^
Male	AIP	2.00 (1.65-2.44)^*^	2.67 (2.17-3.28)^*^	5.94 (4.94-7.15)^*^	7.40 (6.10-8.98)^*^
	T1	Reference	–	–	–
	T2	1.27 (1.13-1.42)^*^	1.38 (1.23-1.55)^*^	1.90 (1.70-2.11)^*^	2.02 (1.81-2.26)^*^
	T3	1.51 (1.34-1.70)^*^	1.76 (1.56-1.99)^*^	2.93 (2.62-3.27)^*^	3.27 (2.91-3.68)^*^
	P-trend	<0.001	<0.001	<0.001	<0.001
Female	AIP	2.45 (1.84-3.26)^*^	2.68 (2.00-3.57)^*^	12.60 (9.55-16.64)^*^	13.36 (10.07-17.73)^*^
	T1	Reference	–	–	–
	T2	1.27 (1.08-1.48)^*^	1.29 (1.10-1.52)^**^	2.17 (1.87-2.53)^*^	2.21 (1.89-2.57)^*^
	T3	1.57 (1.31-1.88)^*^	1.65 (1.37-1.98)^*^	3.92 (3.31-4.64)^*^	4.03 (3.39-4.79)^*^
	P-trend	<0.001	<0.001	<0.001	<0.001
<60	AIP	2.64 (2.01-3.47)^*^	2.82 (2.13-3.73)^*^	6.42 (5.00-8.24)^*^	7.20 (5.58-9.29)^*^
	T1	Reference	–	–	–
	T2	1.27 (1.07-1.50)^**^	1.29 (1.09-1.54)^**^	1.99 (1.69-2.33)^*^	2.08 (1.77-2.44)^*^
	T3	1.70 (1.43-2.01)^*^	1.76 (1.48-2.08)^*^	3.20 (2.74-3.73)^*^	3.43 (2.93-4.02)^*^
	P-trend	<0.001	<0.001	<0.001	<0.001
≥60	AIP	2.22 (1.81-2.73)^*^	2.32 (1.89-2.86)^*^	9.17 (7.51-11.19)	10.05 (8.21-12.30)^*^
	T1	Reference	–	–	–
	T2	1.30 (1.17-1.46)^*^	1.32 (1.18-1.48)^*^	2.02 (1.81-2.25)^*^	2.09 (1.87-2.33)^*^
	T3	1.56 (1.37-1.77)^*^	1.59 (1.39-1.80)^*^	3.39 (3.00-3.82)^*^	3.54 (3.13-3.99)^*^
	P-trend	<0.001	<0.001	<0.001	<0.001

T1: AIP <0.02, T2: 0.02≤ AIP ≤0.23, T3: AIP >0.23.

a Model a: unadjusted.

b Model b: age, DBP, SBP, smoking, drinking, Antihypertensive drugs; ^b’^: Model b’: gender, SBP, DBP, smoking, drinking, Antihypertensive drugs; ^*^ *P*<0.001; ^**^ *P*<0.01.

Based on the variations in characteristics across different stratums, b (stratified by gender) and b’ (stratified by age) were constructed for adjustment purposes.

Pre-DM, prediabetes mellitus; T2DM, type 2 diabetes mellitus; AIP, atherogenic index of plasma; OR, Odds ratio; CI, Confidence interval.

### 3.4 Relationship of AIP with pre-DM and T2DM based on RCS curves

**Figure 2:**
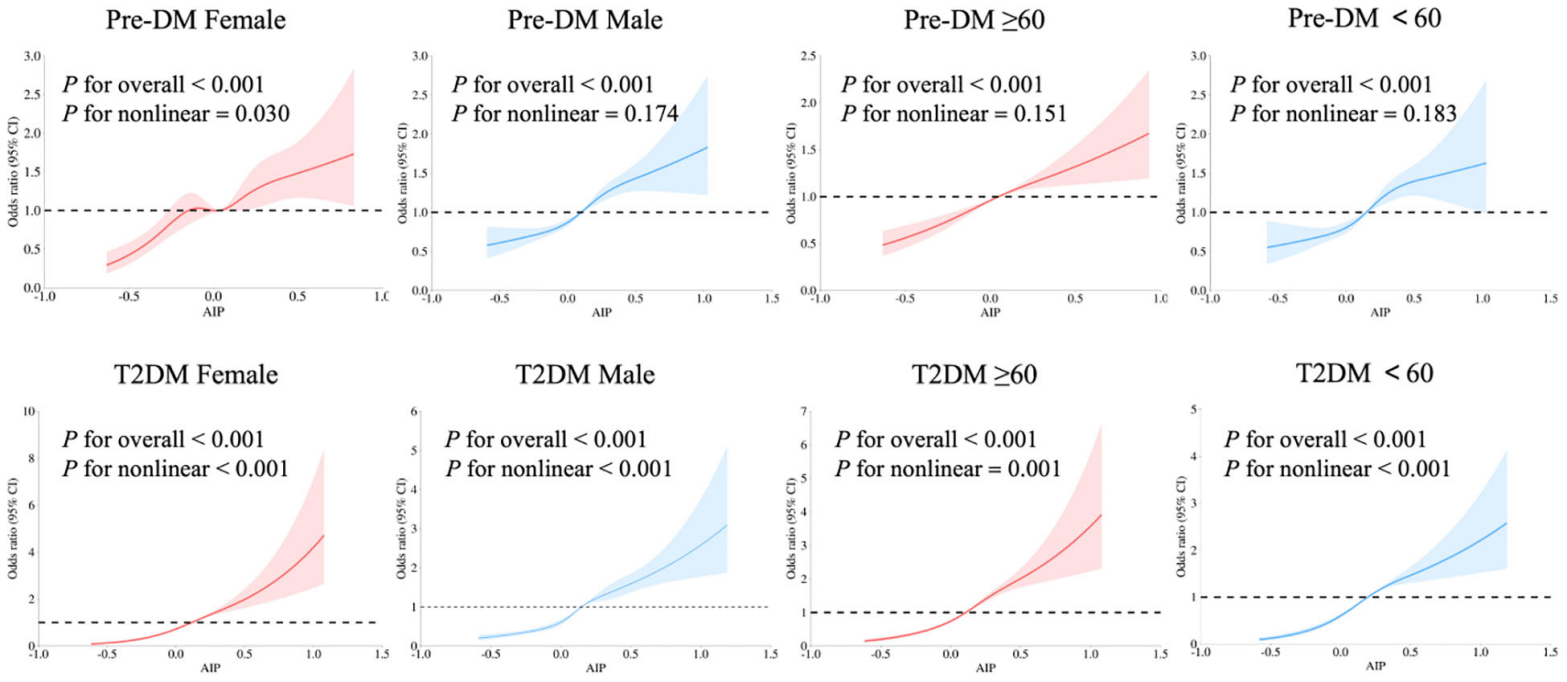
Relationship of AIP with Pre-DM and T2DM in patients with IS of different genders and ages using multivariate-adjusted RCS models. Adjusted for age, gender, SBP, DBP, smoking, drinking, and antihypertensive drugs. The red and blue solid lines represent smoothed curve fits, with the light areas around them indicating the 95% confidence intervals. The OR=1 line is set as the reference line. Pre-DM, prediabetes mellitus; T2DM, type 2 diabetes mellitus; AIP, atherogenic index of plasma; RCS, Restricted Cubic Spline; IS, ischemic stroke.

After full adjustment for confounding factors, there was a nonlinear dose-response relationship between AIP and T2DM in IS patients, which was independent of gender and age (*P* for overall and nonlinear <0.001). Gender differences were observed in patients with Pre-DM: only female IS patients exhibited a nonlinear relationship between AIP and Pre-DM (*P* for nonlinear=0.030). The optimal number of knots for the patients was 5, with the 5th knot located at the 95th percentile, corresponding to an AIP value of 0.461 (**Figure 2**).

## 4 Discussion

Through a retrospective analysis of 18,604 patients with IS, instead of revalidating the academically recognized “macroscopic association between dyslipidemia and IS”, it focused on the association between lipid indices, especially AIP, and abnormal glucose metabolism. This facilitates the earlier identification of patients with abnormal glucose metabolism who have seemingly normal conventional lipid profiles but whose AIP has already indicated potential risks, thereby providing a more precise lipid assessment perspective for the risk stratification of IS patients. This study found that among unconventional lipid indices, the AIP was a key marker for identifying abnormal glucose metabolism, and this association varied with gender and age. Elevated AIP tended to be more strongly associated with Pre-DM and T2DM in female and elderly patients (age ≥60 years) with IS. RCS curve analysis revealed that the relationship between AIP and T2DM presents a non-linear dose-response pattern, while the association between AIP and Pre-DM exhibits a non-linear dose-response characteristic only in the female population. This finding addresses the limitation of previous studies that treated AIP merely as a categorical variable, providing a new quantitative indicator for the early screening of high-risk IS individuals among people with abnormal glucose metabolism. However, it should be noted that this study adopted a single-center retrospective design, and the generalizability of its results to the broader IS population requires further verification.

Pre-DM, T2DM, and Type 1 Diabetes Mellitus (T1DM) all fall within the scope of abnormal glucose metabolism, but there are fundamental differences in their pathological mechanisms. Specifically, T1DM is characterized by autoimmune-mediated β-cell destruction and absolute insulin deficiency (21). As a transitional stage of glucose homeostasis imbalance (19), Pre-DM shares core pathological backgrounds such as IR and lipid metabolism disorders with T2DM. It promotes atherosclerosis and thrombosis by accelerating the formation of advanced glycation end products (AGEs) (22, 23). AIP reflects the ratio of atherogenic lipoproteins to protective lipoproteins (24, 25), with elevated AIP associated with the development of metabolic disorders via multiple mechanisms. For instance, elevated TG stimulates the production of free fatty acids, disrupting insulin signaling pathways in pancreatic α-cells, while reduced HDL-C compromises the β-cells’ protective functions, together forming a vicious cycle of ‘lipotoxicity-insulin resistance’ (26, 27). This mechanism plays a significant role in T2DM and Pre-DM, but it is not a major pathogenic factor in T1DM, which is characterized by absolute insulin deficiency. In view of the above differences and to ensure the homogeneity of the study population, only T2DM patients were included in this study. Additionally, the inflammatory activation (e.g., endoplasmic reticulum stress, adipotoxicity) associated with elevated AIP can exacerbate vascular endothelial dysfunction and is also closely related to increased stroke mortality in diabetic patients (28, 29).

Although some studies have examined the association of unconventional lipid indices with Pre-DM and T2DM, conclusions from such investigations are not consistent (5–7). This may stem from the heterogeneity of study populations. Previous studies did not focus on the specific population of IS patients and mostly used single lipid indicators rather than the comprehensive index AIP; the strength of their associations and gender-stratified results are susceptible to factors such as population characteristics, sample size, and menopausal status. In contrast, this study confirms that AIP has a stronger association with abnormal glucose metabolism compared to conventional lipid indicators. Consistent with previous research, it demonstrates that AIP is a reliable and independent prognostic indicator for long-term monitoring of T2DM patients, with good accuracy in evaluating lipid and glucose metabolism (HR: 1.309, 95% CI: 1.084-1.581; *P*=0.005) (30).

This study found that the association between AIP and abnormal glucose metabolism was more significant in female IS patients. The Framingham Study (31) showed that the risk of IS in females with diabetes increased by 3.6 times compared to males (2.5 times). The NHANES study (12) also confirmed that for each unit increase in AIP, the incidence of Pre-DM and T2DM in women surges by nearly five times (OR: 4.96). In this study, up to 95.5% of the females were postmenopausal, suggesting that the decline in estrogen levels may play a key role. Postmenopausal women often experience reduced TG clearance capacity, decreased HDL-C synthesis, and lowered insulin sensitivity. These changes collectively strengthen the association between AIP and glucose metabolism disorders. A Canadian study (32) showed that the level of IR in postmenopausal women is significantly higher than that in premenopausal women, further supporting the regulatory role of changes in the hormonal environment in the association between AIP and glucose metabolism. Additionally, Gestational Diabetes Mellitus (GDM) is an important risk factor for the future development of T2DM and cardiovascular events (33), and it may be involved in long-term glucose and lipid metabolism disorders by affecting the baseline metabolic status. Future studies could further elaborate on the physiological stages of women and pregnancy-related metabolic backgrounds.

The study also demonstrated that AIP was strongly correlated with abnormal glucose metabolism in elderly patients with IS (age ≥60 years), which matches the metabolic remodeling seen in the context of aging (34). The age-induced anabolic decline and reduced subcutaneous fat storage capacity promote ectopic lipid deposition in organs such as the liver, muscles, and pancreas, thereby inducing lipotoxicity and IR (35, 36). Furthermore, changes in key regulatory factors in glucose and lipid metabolism pathways during aging alter the adipose tissue microenvironment and systemic metabolic homeostasis (37). Previous studies have also suggested that baseline AIP is a reliable indicator for predicting future stroke in middle-aged and elderly patients with abnormal blood glucose (HR: 1.90, 95%CI: 1.52-2.36) (38). Therefore, in the risk stratification of elderly IS patients, AIP can be used as a monitoring indicator for secondary prevention to complement conventional indicators and improve the risk assessment system.

This study also used the RCS model to explore the dose-response relationship between AIP and abnormal glucose metabolism in IS patients, more accurately describing the impact of continuous changes in AIP (39). The results showed a non-linear dose-response relationship between AIP and T2DM, while a linear relationship was observed between AIP and Pre-DM (except in women). This finding provides specific guidance for clinical practice: for example, in the screening of Pre-DM in women, a higher monitoring priority can be set based on the non-linear inflection point of AIP (AIP=0.461 in female). The non-linear pattern observed in women may be related to menopausal endocrine changes, which requires further verification in more studies.

In summary, the AIP, a comprehensive lipid index, when incorporated into IS patient risk stratification tools, significantly improves the accuracy of identifying high-risk IS individuals with abnormal glucose metabolism. Even with normal conventional lipid indices, AIP still accurately indicates potential risks early on. Notably, it is particularly suitable for rapid screening in primary healthcare settings, aiding early intervention.

## 5 Strengths and limitations

This study was a single-center, large sample size retrospective study, which effectively ensured data quality and internal consistency and avoided potential biases caused by differences in different medical institutions. The ample sample size facilitated a more precise evaluation of correlations between variables. The core advantage lies in accurately focusing on the IS patient population, avoiding the limitations of association confounding in general population studies, and verifying that AIP has higher specificity than other lipid indices using a large sample size.

However, as a cross-sectional study, we could not determine a causal relationship between AIP and the occurrence of Pre-DM/T2DM among IS patients. Meanwhile, the lack of long-term follow-up data makes it difficult for us to evaluate the impact of the relationship between AIP and Pre-DM and T2DM on the long-term prognosis of IS patients. In addition, due to the long study period and the limitation of no dynamic monitoring data, potential confounding factors such as Body Mass Index (BMI), socioeconomic status, and dietary information were not fully included, resulting in residual confounding effects. Although excluding users of lipid-lowering drugs and glucose-lowering drugs has controlled the immediate impact of current lipid-lowering therapy on lipid indices, this criterion may also cause the study population to be skewed towards relatively healthy individuals with well-controlled blood glucose and lipid levels, leading to partial selection bias. Future studies can further verify the extrapolatability of the results by including medication users and adjusting for medication factors. In the future, it is still be necessary to rely on prospective cohort studies and in-depth mechanism exploration to further clarify the causal pathways and biological mechanisms between AIP, abnormal glucose metabolism, and IS, thereby providing a more solid theoretical and practical basis for the optimization of stroke prevention and treatment strategies.

## 6 Conclusion

Here, we found that several lipid indices, including TG, HDL-C, TC/HDL-C, TG/HDL-C, LDL-C/HDL-C, Non-HDL-C, RC, RC/HDL-C, and AIP were associated with Pre-DM and T2DM. Among them, AIP is particularly significant, with elevated AIP showing a stronger association with Pre-DM and T2DM. Subgroup analyses revealed a stronger link among female patients and those aged 60 and older. The RCS analysis showed a nonlinear positive dose-response relationship between AIP levels and the incidence of T2DM.

Therefore, the results of this study support the inclusion of AIP as a valuable biomarker in comprehensive health assessment tools for patients with IS. Given the strong association between AIP and Pre-DM and T2DM, especially in females and those aged 60 and older, incorporating AIP into the assessment model can help more accurately identify individuals with IS who are more likely to exhibit potential glucose metabolic disorders and residual lipid abnormalities. In clinical practice, considering the integration of AIP for early risk screening is of positive significance, so as to facilitate the early identification and intervention of high-risk populations.

## Data Availability

The raw data supporting the conclusions of this article will be made available by the authors, without undue reservation.
